# Present and future of extraoral maxillofacial prosthodontics: Cancer rehabilitation

**DOI:** 10.3389/froh.2022.1003430

**Published:** 2022-10-19

**Authors:** Rodrigo Salazar-Gamarra, Salvatore Binasco, Rosemary Seelaus, Luciando Lauria Dib

**Affiliations:** ^1^ Department of Research, Plus Identity Institute, São Paulo, Brazil; ^2^ Centro de Investigación en Transformación Digital, Universidad Norbert Wiener (UNW), Lima, Perú; ^3^ Postgraduation Program in Engineering, Universidade Paulista (UNIP), São Paulo, Brazil; ^4^ The Craniofacial Center, University of Illinois at Chicago, Chicago, IL, United States; ^5^ Postgraduation Program in Dentistry, Universidade Paulista (UNIP), São Paulo, Brazil

**Keywords:** maxillofacial prosthodontics, facial prosthetics, 3D technologies, oral cancer, head and neck cancer, anaplastology

## Abstract

Historically, facial prosthetics have successfully rehabilitated individuals with acquired or congenital anatomical deficiencies of the face. This history includes extensive efforts in research and development to explore best practices in materials, methods, and artisanal techniques. Presently, extraoral maxillofacial rehabilitation is managed by a multiprofessional team that has evolved with a broadened scope of knowledge, skills, and responsibility. This includes the mandatory integration of different professional specialists to cover the bio-psycho-social needs of the patient, systemic health and pathology surveillance, and advanced restorative techniques, which may include 3D technologies. In addition, recent digital workflows allow us to optimize this multidisciplinary integration and reduce the active time of both patients and clinicians, as well as improve the cost-efficiency of the care system, promoting its access to both patients and health systems. This paper discusses factors that affect extraoral maxillofacial rehabilitation's present and future opportunities from teamwork consolidation, techniques utilizing technology, and health systems opportunities.

## Introduction

Head and neck cancer management requires a reconstruction and rehabilitation multidisciplinary plan to transform the original oncological pathology and disability toward restored bio-psycho-social functioning (1, 2). Most head and neck oncology services that want to promote this multiprofessional approach do not have the necessary in-house professionals to address the patients’ broad scope of needs. Therefore, patients are often referred externally or directed to rehabilitation services remotely located (3–6).

The teamwork composition around maxillofacial patients’ needs must include oncology surveillance, systemic physiologic patient condition complications and microbiology, advanced 3D workflow technologies, biomaterials, advanced restorative techniques, osseointegration, and hyper-realistic artistic skills (7–9). All these professional competencies may be concentrated in a system with one or more professionals possessing the competence and legal responsibility necessary for the patient's care. The United States, United Kingdom, and other developed regions are examples of how an education and certification structure has been established for healthcare professionals who must face specializations, subspecializations, and board certification programs to allow patients and health systems to trust their skills and multiprofessional capabilities (10–14). However, this is a specific reality for unique countries that do not necessarily match most parts of the world's public health coverage, needs, and level of education. Furthermore, worldwide professionals cannot justify professions like anaplastology and ocularists in their own countries if their laws cannot support and protect them. Worldwide, dabbler practices are illegal and a public health risk. This is the case when insufficiently trained or supervised lab technicians are treating patients or self-taught people provide care with a self-claimed professional status. They are both dabblers and illegal practitioners facing a severe risk and possible felony. If the country's law does not recognize anaplastology or ocularistry, they have no legal foundation to provide legal patient healthcare in these regions. On the other hand, multiprofessional management empowers individual skills and, under a coordinated intervention and delimitation of responsibilities, allows patients to have a secure rehabilitation process with professionals who exercise their vocation within their defined scope of service.

The American Academy of Maxillofacial Prosthodontics was founded in 1953 when dentists’ first education and training in maxillofacial prosthodontics was of significant concern. In the United States, from 1958 to 1977, 2-year teaching programs were offered. From 1977 to 1984, 3-year programs were offered, and the ADA Commission accredited these on Dental Education (15). Maxillofacial prosthodontists obtain their title after a subspecialization of prosthodontics. This is possible after a dentistry program confers a degree that allows the professional to care for the patient's health as a doctor. The International Anaplastology Association was founded in 1980 as the American Anaplastology Association. Its consolidation as a formal profession in the Unites States arose from wartime necessity. Military hospitals provided care to veterans and identified the need for even more specialized care in both laboratory and clinical setups for the artificial replacement of more complex structures of the face requiring more artistic skills. Thanks to Walter Spohn and Stanford University in 1971, the anaplastology profession started as a formal training program. This 2-year degree course included art and basic sciences, materials and methods, ethics, and business practices. Today, very few places in the world offer formal degree training, usually a 2-year master's program with a previous bachelor’s in art, technology, or other medically related fields (6, 16).

In under-resourced regions, a vicious circle is occurring. The lack of formal education and legal framework maintains professionals without formal training. As a result, fewer professionals remain insufficient to sustain the necessary professional structure within most healthcare systems. Yet, it is a day-to-day reality worldwide among appropriately trained and certified maxillofacial prosthodontists, anaplastologists, and ocularists who are working on solving these real-life problems to serve expanding patient populations.

## Facial prosthetics production

No other body part can reveal feelings, emotions, and character like the face of a person. Therefore, its alteration comes with a solid and intrinsic need to hide facial defects and seek restorative care. Ancient registers support this statement, like the Chinese using resins and metallic parts to hide eyes and faces. Egyptian mummies have been discovered with stone and mosaic replicas of facial parts. Romans documented “eye makers,” “doctors of the eye,” and much more. Restoring anatomy to enable function, cognition reinforcement, and esthetics is a human need (17–20).

Facial prostheses are customized medical-grade devices used to restore severe functional, cognitive, and esthetic alterations to positively impact the patient's daily living activities in a bio-psycho-social way. Three significant steps are well described in the literature to produce facial prosthetics (17, 18, 21, 22). The analog manufacturing process starts with a molding of the facial defect. With the obtained gypsum working model, a sculpture can be fabricated with a thermoplastic material that will mimic the lost anatomy, respecting functional and esthetic principles. Once finished, a mold is created as a negative version of the sculpture. Multiple layers of intrinsically characterized medical-grade silicone are packed accordingly to replicate the patient's skin color. However, in most regions of the world, the prosthetic context requires manufacturing them by analog processes such as manual molding, sculpting, and coloring, as well as using acrylic resin materials, as has been done since the origin of this specialty, among other adaptations of the procedure to the local reality (18, 23–25).

High learning curves exist to exact this technical task and to reduce the chances of a mistake or remakes of the prosthesis. To overcome this artisanal and time-consuming process, specialists have looked to digital technologies to assist or replace some steps in the process, like molding and sculpting (26–35).

### 3D data acquisition

#### Molding processes have been utilizing different 3D image acquisition methods

The first 3D data acquisition trials and digital workflows were performed using MRI data and CT scans because they were the most well-known imaging methodologies to capture the anatomy, becoming more prevalent as cone-beam computed tomography (CBCT) emerged on the market with up to 10% of the radiation dose. Its main advantage is the precision and veracity of the acquired external surface, as well as the possibility of capturing negative areas such as lumens or ears with capricious anatomies. However, nowadays, there is no indication to irradiate a patient with a CT scan for extraoral surface scanning or use expensive MRI technology, both of which lack sufficient image resolution and color information to adequately replicate the level of detail required in a surface scan in facial prosthetics. CT scans have a unique use for extraoral surface data acquisition only when osseointegrated implants are being planned. It can help transport the head's position and allow the designer to mirror the healthy anatomy with just one scan (23, 26, 27, 36–41).

In addition, DICOM images require a thorough segmentation process that can add or remove information from the surface if not handled properly. To segment the facial anatomy, the Hounsfield threshold is used on an appropriate scale. Depending on the application, there are situations where semiautomatic and automatic segmentation tools could be used, but this is at the discretion and responsibility of the treating medical staff. There is no superior tool at present that beats a trained professional with extensive software and anatomy experience performing manual segmentation. Automatic segmentation systems through artificial intelligence are an evolving present. Eventually, automatic systems with artificial intelligence will be sufficiently accessible so that they can be used routinely (42–46).

Laser scanning has been used as an alternative mobile resource to scan extraoral surface structures, with the advantage of not irradiating patients but with the limitation of a noncolored image and limitations for open-eye scanning. Industrial-grade laser scanners were outstanding regarding trueness and precision but were costly and not easily portable given their size. Therefore, they have been replaced by other optic 3D scanning technologies that allow the acquisition of color (UV-map) and were more portable. Recently, the Lidar technique of laser usage has demonstrated potential when combined with optical resources to enhance the best of two data acquisition technologies. However, more studies are necessary to understand its cost-efficiency better (35, 47–49).

The stereophotogrammetry technique is the fastest scanning system because multiple synchronized cameras acquire all of the captures needed in a fraction of a second. It became popular over the last 20 years because of its colored facial scanning. In addition, the standardized hardware presents a low learning curve and reproducibility of its trueness and precision. However, the high-cost investment and the dedicated infrastructure and space needed for a scan rig must be considered. More simplified versions are being developed to reduce space requirements and costs (34, 50–56).

In the past decade, structured light scanners came into the facial prosthesis digital workflows as an active scanning method that creates a 3D stitching process, while the scanner is focused and displaced around the subject. As an optic resource, it produces a colored 3D model. The industry around this technology claimed to be an accessible solution due to the comparison of stereophotogrammetry systems that may cost exponentially more. However, structured light scanners were still not inexpensive enough to become a widespread technology used in most hospitals and under-resourced regions. Additionally, an intermittent flash is not comfortable for an opened eye posture of the patient. Also, the stitching process may accumulate errors in more expansive areas, creating unnoticed errors and holes in the mesh. Finally, the first structured light scanners were not calibrated for facial scanning. Designed primarily for intraoral dental applications, the optical properties may not have the optimal focal distance to obtain the most delicate details of the skin (57, 58).

Lacking the need for special equipment, monoscopic photogrammetry is the most accessible 3D facial surface scanning technique. A unique camera and specific software can be used. Smartphones and open-source software have proven their value in this workflow. When properly used, they have no limit in the computing graphic possibilities, which can manually produce professional and high-resolution 3D images for free. The consideration needed is appropriately controlling the variables with respect to protocols for precision and trueness optimization and a high learning curve to expertly operate the open-source software for rapid data manipulation and satisfactory results (31, 59–66).

**Figure 1:**
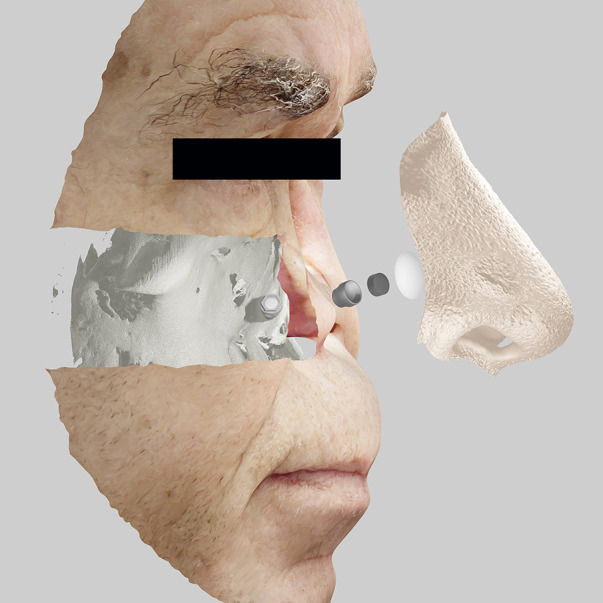
Integration of medical images with planning of implants, components, and prosthetic design for extraoral bucco-maxillofacial rehabilitation on implants.

Face scans with techniques such as monoscopic photogrammetry, precisely executed, are getting closer in precision and accuracy compared to tomographic methods. Even so, in cases such as the evaluation of craniofacial implants, there is an opportunity to compose 3D scans with those of surface scans. In this way, it is possible to obtain the best advantages of multiple systems and technologies in a more digital and integrated treatment. There is no single best technology for every case. It is necessary to intelligently use all the available resources that the patient and the context allow (Figure 1) (67–73).

### 3D modeling

Independent of the chosen technology for 3D facial scanning, the virtual 3D model needs to be manipulated within a CAD program. The standard tools necessary are duplicating, cutting, transforming, sculpting, and Boolean operations, which can be performed in almost any CAD software, apart from whether it is freeware like Meshmixer or high-cost commercial license software like Zbrush. Of course, previous user experience, learning curve, and user interface are individual criteria contributing to the designer's software selection. On the other hand, professional open-source software like Blender allows senior designers to take advantage of much more complex operations like modifiers, physic simulation, animations, merging, CMYK color model data in virtual reality modeling language (VRML) exportation, and others. The +Plus ID Institute programmed the first facial prosthetic design software as an add-on in Blender, which can be used for free (62, 63). Also, some algorithms are being developed to automatically detect the coloring of the facial prosthesis thanks to a deep artificial neural network approach to coloration in a facial prosthesis (74).

### 3D digital fabrication

Different digital manufacturing technologies have been described for facial prosthetic digital workflows, from subtractive techniques of wax, metals, and polyether ether ketone (PEEK) to additive manufacturing with fused filament fabrication (FDM), stereolithography/liquid-crystal display/digital light processing (SLA/LCD/DLP), polyjet, selective laser sintering (SLS), selective laser melting (SLM), and, more recently, silicone 3D printing (31, 63, 75–77).

FDM has been the most popular 3D printing technology since the Stratasys patent release. The thermoplastic filament is the most accessible 3D printing material that can replicate the macroanatomy of a facial structure but has a limitation on the microanatomy due to the evident layers and its staircase effect. On the other hand, all resin 3D printing technologies (SLA, LCD, DLP, Polyjet) have demonstrated their ability to reproduce the most delicate details of facial skin microanatomy characteristics (78).

Medical-grade silicone 3D printing is the most desired and expected technology consolidation. Some efforts have been made with success, although challenges still exist (79–83). However, voxel-colored Polyjet 3D printers may have a future in this realism and reliability where the +ID institute enabled the translation of color from smartphone captures into a 3D printed colored orbital prosthesis used by the patient with no complications (64).

## Discussion

The future is technological and in teamwork. The ideal coming landscape for maxillofacial prosthodontists, anaplastologists, and ocularists is having worldwide opportunities for formal and accessible education. This will allow future professionals to fulfill the health system and patient needs, working together in an integrated health system with patient coverage of their advanced and accessible treatments. The next generations of 3D image acquisition systems bring an automated and self-calibrated, self-scaled 3D model that can mix more than one technology and dynamics with no high cost in a mobile and portable scenario. The next advances in 3D modeling of facial prostheses will make possible an open-source automated design created by artificial intelligence that can recognize the patient's anatomy and replace the missing part with self-created 3D meshes. The future of the 3D manufacturing process of the facial prosthesis is the final and direct 3D printed prosthesis with the high manual capacity of a gold standard exhibited by the most skilled prosthetists.
